# Editorial: Non-coding RNAs in gastrointestinal and gynecological cancers: New insights into the mechanisms of cancer therapeutic resistance, volume II

**DOI:** 10.3389/fcell.2023.1161164

**Published:** 2023-02-14

**Authors:** Peixin Dong, Lei Chang, Junming Yue

**Affiliations:** ^1^ Department of Obstetrics and Gynecology, Hokkaido University School of Medicine, Hokkaido University, Sapporo, Japan; ^2^ State Key Laboratory of Radiation Medicine and Protection, School of Radiation Medicine and Protection, Collaborative Innovation Center of Radiation Medicine of Jiangsu Higher Education Institutions, Medical College of Soochow University, Suzhou, China; ^3^ Department of Pathology and Laboratory Medicine, University of Tennessee Health Science Center, Memphis, TN, United States; ^4^ Center for Cancer Research, University of Tennessee Health Science Center, Memphis, TN, United States

**Keywords:** non-coding RNA, microRNA, long non-coding RNA, circular RNA, chemoresistance, cancer biomarker, gastrointestinal cancer, gynecological cancer

Non-coding RNAs (ncRNAs) are increasingly being recognized as key participants in a broad variety of biological processes. It has been shown that ncRNAs are important in predicting patient survival as well as regulating the resistance to chemotherapy, radiotherapy, targeted treatment, and immunotherapy (Wang et al., 2019; Chen et al., 2022). Excitingly, numerous ncRNAs are now being investigated in clinical trials for their potential as indicators of response to cancer treatment (Chen et al., 2022). In this Research Topic, we addressed the functional relevance of ncRNAs in the resistance to the treatment of gastrointestinal and gynecological cancers.

Hu et al. created and validated a prognostic signature for ovarian cancer based on histone acetylation-related long non-coding RNA (lncRNA). They used transcriptome data from the Genotype-Tissue Expression Project and the Cancer Genome Atlas (TCGA) to create a risk profile for ovarian cancer using 14 histone acetylation-related lncRNAs. The newly developed risk score model may be more sensitive and accurate in predicting the outcome of ovarian cancer patients. Overall, this research reveals that the risk signature, which consists of 14 histone acetylation-related lncRNAs, might be used as markers and prognosis predictors in ovarian cancer patients.

Early cancer detection enables efficient treatment. *QKI* is believed to be a tumor suppressor gene, however, its diagnostic utility in colorectal cancer has not been investigated. Zhang et al. used various cancer tissue and cell line methylation data to determine the methylation status of QKI. The diagnostic performance of QKI was evaluated using test sets from the Gene Expression Omnibus (GEO) and TCGA. In 31 tumor samples, all the *QKI* promoter CpG sites exhibit colorectal cancer-specific hypermethylation. The authors investigated the diagnostic performance of ten CpG sites in identifying colorectal cancer or adenoma from normal tissues, and they determined that a total of ten sites could discriminate between the two. In addition, they discovered that the methylation of the QKI promoter occurs early in the formation of colorectal cancer. The authors conclude that methylation of the *QKI* promoter is a colorectal cancer-specific signal with considerable potential for improving the early diagnosis of colorectal cancer.

Exosomes are released by various types of cells in the tumor microenvironment (TME) and engage in a variety of tumor biological activities. NcRNAs encased in exosomes and released into the TME have been implicated in cancer and development, as well as acting as essential intracellular communication mediators. A review by Chen et al. summarized the role and mechanisms of exosomal ncRNAs in mediating the modulation of both tumor cells and immune cells. The exosomal ncRNAs secreted by tumor and stromal cells show potential capabilities to regulate tumor cell growth, progression, metastasis, drug resistance, and anti-tumor immune response. This review will hopefully stimulate more research into the potential of exosomal ncRNAs as diagnostic, prognostic, and therapeutic biomarkers for cancer.

In gastrointestinal cancer, ncRNAs have been demonstrated to regulate radiosensitivity. A review by Li et al. discussed the complicated roles that ncRNAs play in gastrointestinal cancer radiotherapy. NcRNAs can function as radiosensitivity enhancers or radioresistant inducers in gastrointestinal cancer by targeting genes. The predictive value of ncRNAs in response to radiotherapy was also recognized, and individualized radiotherapy might be guided by ncRNAs. This study laid the foundation for introducing ncRNAs into gastrointestinal cancer radiotherapy as response predictors, radiosensitivity enhancers, and radioresistance inducers.

Ghafouri-Fard et al. covered the role of TMPO-AS1 in carcinogenesis and discussed its potential as a diagnostic marker for certain forms of cancer. The frequent overexpression of lncRNA TMPO-AS1 in human tumors has been reported in many cancers and was correlated with worse patient survival. In cancer cell lines and xenograft models, suppressing TMPO-AS1 reduces malignant behavior. Moreover, TMPO-AS1 silencing has been demonstrated in preclinical trials to improve sensitivity to paclitaxel and docetaxel in endometrial and breast cancers, respectively. It would be important to investigate the role of this lncRNA in tumor chemoresistance further.

Importantly, the complexity of cancer tissues may produce contradictory findings. Future development of a single-cell sequencing method for ncRNAs will provide answers to queries addressing the intratumoral heterogeneity of ncRNAs (Chen et al., 2022). In addition, multiple studies have confirmed the presence of mature miRNAs in the nucleus, and the transport pathway between the nucleus and cytoplasm has been described (Liu et al., 2018). The subcellular function of ncRNAs in resistant patients has to be fully investigated (Chen et al., 2022).

Together, these articles in this Research Topic provide further evidence that ncRNAs play a critical role in mediating cancer therapeutic resistance and suggest that studying ncRNAs in gastrointestinal and gynecological cancers will yield useful insights that could be applied to the design of more efficient diagnostic and therapeutic strategies.
